# Playbook of Subnational Illiberalism: Autocrats Face the Opposition-led Local Governments

**DOI:** 10.1007/s40803-022-00184-8

**Published:** 2022-11-21

**Authors:** Mariam Begadze

**Affiliations:** Central European University, Budapest, Hungary

## Abstract

Recognizing the growing tensions between autocrats in the center and opposition-led local governments in Hungary, Poland and Turkey since 2018–2019 local elections, the article contributes to existing literature on illiberal democracies with a subnational portion of illiberal playbook. Tactics identified through the detailed study of the European context and brief review of Latin American experience leaves us with the following categories in the playbook: abuse of (existing) supervisory and accountability mechanisms; generating of financial vulnerability; centralization (outright and indirect) and deconcentration. Each of these categories assemble various means evolving through application and reinterpretation of traditional rules pertaining to local government, as well as crisis-induced innovations. While the Polish account carries the optimism still that antecedent robust guarantees and popular support matter even when illiberals rule the center, the playbook proved successful in Hungary and Turkey. Although certain incrementalism stayed as the most vulnerable actors were the first victims of soon-to-be normalized measures, crisis in Hungary and Poland did stretch the limits to the point that ulterior motives of undermining opposition-led local governments became publicly observable. Reflecting on this phenomenon, in the end, the article poses a theoretical question whether such pretextual instrumentalization of law can itself be judicially manageable, at least in situations when clear political opponents are targeted.

## Introduction

This article explores a somewhat neglected aspect of subnational illiberalism, when illiberal governments in the center concentrate power to undermine opposition-led local governments. Rather than presenting the liberal enclaves as a sign of reinvigorated national democracy and rule of law, this reality is seen as an incentive for central illiberal regimes to deploy new autocratic tactics for recentralization of power, continuing to erode rule of law alongside other tactics already in the illiberal playbook. Incentives to undermine political opponents are clear and ample, including fear of effective government on the local level that can and in some places has become a platform for building support for an electoral victory on the national level (Lucardi 2016; Farole 2021). Although concentration of power in the central government is no negligible tool for the perpetuation of power, unlike the dismantling of separation of powers, it is a far more tolerated institutional design from the point of constitutional law. The dependent judiciary is simply incompatible with rule of law and separation of powers, while dependent local governments seem to be less scrutinized despite some binding standards on local autonomy on the European level (Boggero 2017).^1^ In this context, Illiberal techniques against central institutions have monopolized the attention. As Jakli and Stenberg observe ‘[w]hile numerous studies consider the roles that media consolidation, court-packing, and economic crises have played in Hungary's democratic decline since 2010, none have considered the subnational mechanisms driving illiberalism’(Jakli and Stenberg 2021). They themselves address a specific type of subnational illiberalism, that used to undermine opposition when it is not governing on the local level, by limiting opportunities for political contestation and oversight.^2^ Much academic literature has discussed the reverse scenario of autocratic enclaves at the subnational (state) in otherwise democratic federal (Behrend and Whitehead 2016; Gibson 2013) or supranational regimes such as EU, for which Hungary and Poland themselves are ‘local pockets of autocracy’. Kelemen pointed to structural weaknesses of federal and supranational structures such as internal political competition and inadequate conditionality relating to the provision of funding in the EU as responsible for toleration of such enclaves (Kelemen 2017, 2020). In this special issue Hernández and Closa discuss a particular case of undermining rule of law from within discussing the breaches thereof by political leaders in the Catalan secessionism movement (Hernández and Closa 2022). This article is contributing to the discussion about regime variance within the state from a perspective of national illiberal tactics against local opposition enclaves. Such tactics have already been discussed in Latin American context, as summarized below (Dickovick and Eaton 2013). This article will analyze the presented problem in the European context.

Case studies chosen for this inquiry are Hungary, Poland, and Turkey. These states conveniently juxtapose illiberal rulers against new enclaves of opposition-led local governments following recent victories in local elections held in October 2018 in Poland, March 2019 in Turkey and October 2019 in Hungary. These states share similarities, such as their unitary form of government and prior recentralization tendencies, whilst they still differ as to the degrees of local autonomy guarantees and its legitimacy among the public at the point of illiberals assuming power, with the Polish local autonomy standing out as the most robust on both counts. Freeman argues liberal enclaves in otherwise authoritarian states can be sustained if two conditions of subnational mobilization and international backing are met (Freeman, 2020). It is too early to judge this for Hungary, Poland and Turkey. Instead of speculating about the eventual survival of these enclaves, this paper tracks the illiberal playbook specifically deployed against them, solid understanding of which must come before legal and political solutions are conceptualized.

Apart from identifying a playbook of subnational illiberalism, the analysis eventually leads to several observations confirmed by cases from both continents. 1. many of the tactics are enabled by structural (perhaps some of them even inevitable) weaknesses in the accepted norms of local government; 2. crisis situations bringing about previously unavailable pretexts (Stenberg et al. 2022; Grogan and Donald 2022) and unlocking exceptional rules of states of emergency (Lührmann and Rooney 2021) may stretch the limits to an extent that ulterior motive of undermining opposition-led local governments become publicly observable. Similar dynamic was observed by Grogan and Donald in a multi-country study of the pandemic and rule of law, stating the pandemic ‘had the effect of accelerating pre-existing trends and exposing the true character of regimes’, while autocratising states such as Hungary and Turkey in particular, ‘seized upon the virus as a pretext to entrench power at the centre’ (2022, 474). 3. Given the above points, the logic of subnational illiberalism is incremental, opting for subtle legalistic and technical means of weakening autonomy of local governments, whilst crisis may radicalize the measures and exemplify the ulterior purposes, as the law remains an instrument. Certain incrementalism also stays. As the examples from both Turkey and Hungary will illustrate even in crisis situations subnational illiberalism first gets tested against the most vulnerable to be normalized for a more across-the-board application later.

Use of existing weaknesses in the constitutional system and pretextual rulemaking has been characteristic to illiberal democracies, captured in the terms ‘autocratic legalism’ and ‘ruling by cheating’ used by Scheppele (2018) and Sajó (2021) respectively. More recently, Pirro and Stanley categorized illiberal playbook in Hungary and Poland as ‘forging, bending, breaking’ of law presenting it as an incremental process where the ‘the art of finding the cracks’ in the pre-existing system is learned ‘from emulation of other cases’. According to them, illiberal regimes more often than not follow the letter of the law, while contradicting its spirit by ‘reinterpretation or disabling of existing legislative constraints’. (Pirro and Stanley 2022, p. 89–90). As Sajó puts it ‘illiberal democracy knows how to behave and, […] it cheats only to the extent that is necessary’ (Sajó 2019). This is besides the strategy to achieve systemic, aggregate anti-constitutional effect through fragmented otherwise tolerated weaknesses, often borrowed, with the minimum political cost possible (Scheppele 2013, 2018). The analysis in this article confirms general validity of these theoretical frameworks revealing nuances characteristic to undermining enclaves of liberal government besides those already known about undermining of accountability structures on the central level.

To enable such an analysis, the article will first briefly overview the experience of undermining opposition-led local governments in Latin America, and then will discuss the European experience in more details before the playbook of tactics for the weakening of opposition-led local governments is laid out. I will draw attention both to more gradual trends of (re)centralization and later targeting of opposition-led local governments with the crisis-related pretexts.

## Praxis of Subnational Illiberalism

### Latin American Origins of Subnational Illiberalism

Cursory analysis of Latin American experience reveals that, as a rule, illiberal center follows the subtle, incremental and technical/legalist approach in its efforts to undermine opposition-led local governments. Often this is done without breaking the law or changing it as some of the weaknesses of local government are embedded in existing legal regimes (Kersting 2009, 107–108).^3^

Commonly, the center in Latin America claimed back authority in certain areas (public schools in Argentina since 2006; ports and airports in Venezuela since 2009) or created deconcentrated bodies with overlapping functions (in the area of managing all public investments in Peru since 2000) (Dickovick and Eaton 2013, 1456, 1459). The latter tactic took its radical form in Venezuela. A series of legislative changes adopted by 2009, upon the initiative of the pro-Chávez bloc in the legislative body created a nationwide network of so-called Communal Councils operating under the new central institution—the Federal Council. Displaying the incremental nature of decentralization, this followed after the 1999 Constitution abolished the Senate, and the recentralization reform had narrowly failed in a referendum in 2007. As Freeman notes a ‘strikingly similar duplication of local government unfolded under Viktor Orbán’ to be discussed below which again evidences how illiberal playbook including a subnational one travels (Dickovick and Eaton 2013, 1459, 1462; Freeman 2020, 44; Kestler 2018, 635).

Through technical modification of rules on revenue-sharing, states in Latin America have also changed the balance of available funds in their favor. Mostly, such changes had across-the-board application.^4^ An exception was the creation of off-budget funds under direct presidential control of Chávez (filled with oil exploitation and general tax surpluses) enabling selective distribution of revenues to be followed by punitive withholding of funding (Kestler 2018, 534). Although also universally applied, Venezuelan government also took a radical step of funding the new deconcentrated communal councils at the expense of subnational governments (Dickovick and Eaton 2013, 9).

As Dickovick and Eaton summarized both political and fiscal recentralization in Latin America had ‘a pronounced tendency towards indirect approaches and technical changes that increase central leverage incrementally over time’ (Dickovick and Eaton 2013, 1456). As they explain ‘[o]perating at times under the radar screen and eschewing the type of directly confrontational approaches that might ignite the unified opposition of subnational elected officials, national politicians and bureaucrats have demonstrated cunning, versatility, and persistence in their attempts to restore the centre’ (Dickovick and Eaton 2013, 1463).

As will be shown from the detailed discussion of practices in Hungary, Poland and Turkey below, illiberal tactics on the subnational level also get emulated (e.g., network of deconcentrated bodies in Hungary borrowed from the Venezuelan counterpart), and share incremental, subtle and technical/ legalist nature.

### Incremental Centralization During Normal Times in Hungary, Poland and Turkey

Like most Latin American countries, all three unitary states discussed in this article went through decentralization Zeitgeist before centralization push reemerged as the hybrid regimes consolidated power. Addressing the communist-type centralization, Poland (Monitoring Committee 2019, para 10),^5^ and to a lesser extent, Hungary undertook decentralization reforms in 1990s. As observed in the literature, despite significant processes of institution-building on the local level in Hungary, ‘overall, the power structure has remained centralized’ (Pálné Kovács 2013, 189). Due to internal problems of fragmentation, lack of resources and ineffective functioning, by the time Orbán came to power in Hungary, centralization reforms had acquired legitimacy in the political and professional circles (Rajca 2020 136). Erdoğan had first initiated decentralization reforms alongside neo-liberal policies in the early 2000s to reverse that after the 2010 constitutional referendum. Many argued the decentralization reforms were more about the transfer of responsibilities than increasing fiscal and political autonomy in Turkey (Savaşkan 2021, 206). Although some areas were taken over completely, for instance, public schools and hospitals in Hungary (Hajnal and Rosta 2019), disciplinary supervision of the municipal staff in Turkey (Monitoring Committee 2022, para 98), much of the recentralization in Turkey (Savaşkan 2021, 212–214) and Hungary (Hajnal and Rosta 2019) took place through the creation of parallel deconcentrated bodies that supervise local authorities sometimes even overriding their decisions.^6^ In Hungary, the changes to local autonomy started with the new Constitution. Among other changes, such as creation of the network of deconcentrated bodies as in Venezuela, the new Constitution, did not classify local government property as a separate category, rather as a type of national property (Rajca 2020). That the constitutional status of self-government property was undermined in this manner, was relevant for the measures adopted during COVID-19 pandemic and its consideration before the Constitutional Court. More details of how recentralization proceeded in Hungary and Turkey will be discussed below.

Unlike Hungary and Turkey, Polish local governments proved resilient, arguably due to antecedent (Slater and Simmons 2010)^7^ more robust public support for local autonomy—72% approval in 2016 (Freedom House 2017; Rajca 2020), guarantees in the constitution including access to judicial remedy for breaches of autonomy,^8^ power to set levels of local taxes, and strict regulation of legal supervision through the law (Monitoring Committee 2019, paras 91–96, Sześciło, 2019), as well as developed case-law compared to weak legal standards, including weak access to courts of local governments in Turkey^9^ (Monitoring Committee 2022, paras 31, 51, 248) and Hungary (Hajnal et al. 2021, Monitoring Committee 2021, paras 4, 253–254).^10^ The most important legislative change adopted in June 2017 increasing powers of a central agency tasked with financial supervision over self-government spending in Poland was vetoed by President himself with arguments referring to constitutional guarantees of self-government (Sześciło, 2019). The change if implemented would have increased the powers of the central government in determining the composition of the financial supervision body (competitive procedure was substituted with the discretion of Prime Minister in appointments), also it would have extended the scope of review beyond legality to include cost-effectiveness and reliability review at least when it came to loans, credits and municipal bonds. These changes would have made it easier to dismiss local politicians under investigation (Monitoring Committee 2019, paras 99–100). Some centralization measures also succeeded in Poland, for instance, in 2016, the center acquired the upper hand in designing the school system, although the management of the public schools remained the competence of local governments. Centralization also extended to competences such as regulating tariffs for water and sewage disposal, investments in sports development, issues of farmers’ support, designation of voting precincts. By changing the composition of supervisory boards of autonomous regional environmental protection funds (reducing members from the majority to one out of five), centralization was achieved indirectly in this field of implementing environmental protection programs as well (Sześciło 2020; Rajca, 2020; Monitoring Committee 2019, paras 124–125). Existing powers of legal supervision were also more actively used in the last period (Monitoring Committee 2019, para 213). Despite some criticisms concerning emerging signs of centralization, justified by the government as an effort to build ‘uniform standards of access to services and benefits’ (Rajca 2020, 142), without a qualified majority, central government could not amend guarantees for local autonomy on the Constitutional level, neither did the changes on the legislative and policy level lead to a major shift back to recentralization, as in Turkey and Hungary. Polish illiberals seem to opt for a different path, whilst mostly tolerating the institutional robustness of local government. In September 22, 2022, aware of dropping rating of opposition, lower house of Poland’s parliament (Sejm) voted in favor of delaying next local elections to spring of 2024 instead of autumn of 2023, presented as a necessary organizational measure, namely, to avoid coinciding with the parliamentary elections (Ptak 2022). This would not be the first time that the Polish Parliament attempts to increase its prospects of victory by determining the timing of elections (holding it as planned despite the pandemic) considering the polls (Ziółkowski 2020; Sadurski 2020).

Nevertheless, even in Poland, the Constitution alongside providing robust guarantees for local autonomy also entails mechanisms that could be used against opposition-led municipalities. The Polish Constitution foresees the possibility to dissolve a local government unit, but only with the decision of Sejm upon the request of the Prime Minister if it has flagrantly violated the Constitution or a statute (Monitoring Committee 2019, para 210). Similarly, the legal framework before recentralization already entailed elements prone to abuse in Turkey. An institution of administrative tutelage, namely that central authorities control the activities of local authorities covering both legality and efficiency issues,^11^ enshrined in the Constitution is the best illustration (Monitoring Committee 2022, para 4 (d)). It is noteworthy that the administrative tutelage is so much embedded in the political tradition of the country that most of the local representatives do not question this feature of the system, at least as a matter of principle (Ibid, para 148,). In this administrative tutelage system, inspection conclusions about gross negligence in the municipal services, constituting a risk to public health, peace, and safety, if confirmed by courts, can eventually lead to a substitution of a mayor by a governor (Ibid, paras 151–156, 245, 259) Similar mechanism exists for municipal and provincial councils that fail to perform their functions or pass resolutions on political matters, the latter (introduced in 2005) being a particularly vague category prone to abuse as also evidenced in practice (Savaşkan 2021, 213).^12^ In such cases, the Ministry can suspend council meetings until the decision is made by the Council of State. The decision to approve dissolution is to be followed by a new election to constitute the new council (Ibid, paras 158–160). As will be seen some of the elements of these rules, later appeared in radical measures on removal of mayors, but without the safeguards such as judicial supervision present here.

Within the same administrative tutelage framework, various ministries and central government agencies have power over municipal decisions on specific issues in the form of supervision, approvals, deferments etc. (Ibid, para 157). Another mechanism upheld by the Constitutional Court allows for the transfer of properties of municipalities and of special provinces to the Ministry of Interior for reasons of security (Ibid, para 184). As will be seen, a similar possibility, but with economic rationale, was created during COVID-19 pandemic in Hungary. What the Turkish institution of administrative tutelage illustrates is that often uncontroversial aspects of local government system provide ample room for abuse against opposition-led local governments, while existing rationales behind them can help legitimize the further stretching of the control.

Levelling the control of Turkey’s administrative tutelage system and emulating Venezuela during Chávez, In 2011, Hungary introduced nation-wide network of supervisory bodies (county government offices). Within the scope of its legality review power, these bodies can take steps with the Hungarian State Treasury to suspend or withdraw a certain portion of support from the central budget as specified by law, initiate disciplinary proceedings and sanction local governments with fines, file a court motion to terminate the mandate of a mayor who commits repeated legal violations, recommend to the Government to dissolve assemblies operating in contravention of the Fundamental Law, which can be done after obtaining the opinion of the Constitutional Court (Monitoring Committee 2021, para 83). This mechanism in Hungary also confirms that possibility of targeted undermining of opposition-led municipalities is embedded in otherwise ‘tolerated’ constitutional framework of local governments.

Another development both in Turkey^13^ (Monitoring Committee 2022, para 98) and Hungary^14^ (Monitoring Committee 2021, para 95) is the change of composition in bodies previously controlled by local governments through adding representatives of the central government that guarantees that the latter can veto decisions.

What is also widespread are the mechanisms of conditional (criteria-based) financing, task-based subsidies or other types of discretionary support to local governments as in Hungary (Rajca 2020, Monitoring Committee 2021, paras 74–75, 229–230), also loans that require approval of the center which give broad leeway for selective application, as in both Hungary (Hajnal and Rosta 2019, Monitoring Committee 2021, paras 69, 72, 233), and Turkey (Savaşkan 2021, 212, Monitoring Committee 2022, para 213). When intermediary bodies are involved in distribution of such funds, their composition rules tend to be changed to increase the control of the central government. For instance, in Turkey right after local elections in March 2019, in which opposition won local seats, changes were made to law to increase President’s power over İlbank (previously Bank of Provinces), the institution that directs intergovernmental transfers to subnational governments and provides loans, as well as the Directorate of Strategy and Budget in relation to its authority of providing financial aid to the municipalities (Elicin 2020). Soon, in September 2019, when a loan was needed for finalizing the metro line construction, state banks refused to deal with the Istanbul opposition mayor (Öktem 2021). Such discretionary mechanisms are embedded and normalized in the center-regional relations. When own revenues constitute a small part of the total budget as it is Hungary (Monitoring Committee 2021, paras 216–219), and to a lesser extent, in Turkey (Monitoring Committee 2022, paras 178, 186), such discretionary mechanism of central distribution of funds become more problematic. The tactics against subnational governments became more direct during crisis situations as post-coup period of 2016 in Turkey and global pandemic of COVID-19 since 2020, which conveniently brought about new pretexts for the central governments. Whilst opposition already had significant representation on the local level since 2014 elections in Turkey, the capital and several other big cities also fell under opposition control after 2019 elections. In Poland and Hungary, liberal enclaves in an otherwise illiberal state emerged after 2018 and 2019 local elections, respectively. The section below will turn precisely to crisis-related measures against opposition-led local government in Turkey and Hungary. In Poland no major change happened in relation to local governments despite the ruling party using the pandemic opportunistically in other areas (legal access to abortion, criminalization of sexual education, election regulations) (Guasti 2021; Hajnal et al. 2021). Following the pandemic, the opposition has even united around the demand for more autonomy and powers of local governments (Sieniawski 2022). As pandemic or other crisis had not produced significant curtailment of local autonomy in Poland, the discussion below will focus on Hungary and Turkey.

### Subnational Illiberalism During Crisis in Turkey and Hungary

The most drastic measures against opposition-led local government—denial and suspending (de facto termination) of local elected  mandates without court approval—come from Turkey. These mechanisms combined with other aspects of the legal system (e.g. loose definition of the crime of terrorism) allowed effective revocation of election results. This measure against elected local officials in Turkey by itself marks a new chapter in the life of hybrid regimes. Through this legal change, the law is not only used to tilt the election playfield but to completely annul local elections. Through *post factum* annulling of election results not only rule of law, but democracy in its minimal sense is compromised. These developments in Turkey have attracted harsh criticism from the European Parliament (2019), Council of Europe Parliamentary Assembly (2019), the European Commission for Democracy through Law (Venice Commission, 2017b), Committee on the Honouring of Obligations and Commitments by member States of the European Charter of Local Self-Government (Monitoring Committee 2022).

The measure on de facto termination of local elected mandate originates in a pre-pandemic local crisis of an attempted coup in Turkey. In 2016, following the declaration of a state of emergency, a decree created the basis to replace local elected officials with government-appointed trustees if the former were accused of a terrorism-related criminal act. According to a prior law adopted in 2005, if the mayor’s position became vacant including by decision of the Ministry of Interior to remove them, mayors were to be replaced by other elected officials chosen by the municipal council. In the new scenario, trustees run the municipality, while the municipal council is prohibited from convening on its own motion. This amounts to de facto dissolution of the council along with the removal of mayors (Monitoring Committee 2022, paras 55, 279, 296). It is important that this measure had previously failed to pass through ordinary legislation and became a possibility only during a state of emergency despite widespread criticism, even from the Justice and Development Party (AKP) members themselves. By the time the emergency was lifted in 2018, the new rule in the emergency decree had turned into a permanent legal regulation. Although state representatives claimed that the absence of conviction would lead to reinstatement, mayors are practically never reinstated either because court decisions are not made before the term expires, or if they are, even if it is exonerating, new charges are presented (Venice Commission, 2017b, para 61, Monitoring Committee 2022, paras 268–272). The legal analysis of these measures both undertaken by the Venice Commission and the Monitoring Committee does not omit to mention the importance of the vague and loose definition of ‘terrorism’ in criminal legislation, which when considered in conjunction with these measures exacerbates the risks of abuse. This exemplifies a phenomenon noted above when the systemic, aggregate anti-constitutional effect is achieved through fragmented weaknesses of law (Scheppele 2013).

As for the implementation, although Kurdish political parties had nothing to do with the coup, between 2016–2018, based on this rule, 94 out of 102 Kurdish mayors were removed following investigations on the grounds of an overly broad definition of ‘terrorism’ (Whiting and Kaya 2021). The Turkish Constitutional Court did not find grounds in the individual appeals that argued presumption of innocence was at stake due to removals before conviction (Stockholm Center for Freedom 2022). During the pandemic, the practice of removing mayors continued. In September 2020, the last big city mayor was removed following around a hundred of other mayors from the Peoples’ Democratic Party (HDP) before him (POLITICO 2020). Apart from HDP mayors who are certainly the main target, removals have occasionally extended to a few from the main opposition party—Republican People's Party (CHP) as well (Parliamentary Assembly 2019; Ahval 2020). Nevertheless, while use of these measures became normalized against pro-Kurdish politicians arguably due to tolerated hostility towards Kurdish separatism in general, the center seems to be more careful towards the main opposition party. In the meantime, justifications for removals have become detached from the security context as well. As Tutkal observes, trustees now increasingly refer to more effective government through better links with the central government and less to the security threats posed by substituted Kurdish mayors (Tutkal 2021).

The practice of removing mayors after 2019 elections emerged in a new form when the mandate was denied to elected local officials without any new investigations against them and even though they were not disqualified before the elections. Elected officials were denied the mandate by the Supreme Election Council (SEC) once they assumed the responsibility to give it to those who came second (and lost) in the elections—the ruling party, on the basis that the candidates had been dismissed during the state of emergency due to them being banned from public office in several decree laws adopted during that period (Monitoring Committee 2022, paras 83–84). As the Venice Commission emphasized, there is no provision in Turkish law that would explicitly give the SEC the competence not to hand over the election certificate to an elected candidate in cases where ineligibility is invoked after the election,^15^ also, the practice of SEC was previously consistent pronouncing that a disqualification ground—‘those who are prohibited from public services’—meant only situations when court decisions imposed that sanction. Indeed, this was the position taken by SEC unanimously in 2018 when rejecting objections against several candidates for parliamentary elections who had been prohibited from being employed in public service by emergency decrees. That the position changed with regard to local municipalities and especially pro-Kurdish candidates is again indicative, that the radical measures are likely to be used against more vulnerable local players presumably to be normalized and used on a larger scale after. As SEC decisions cannot be appealed before the Constitutional Court, there is not even a theoretical legal avenue to remedy this domestically (Venice Commission, 2020, para 11).

Importantly, the Venice Commission and Monitoring Committee have hinted at the issue of pretext in its reports discussing the mechanism on removal of mayors. The Venice Commission stated that introduction of permanent changes was all the more questionable considering the amendment was on the agenda earlier in 2016 as part of a bill, which could not be passed due to the opposition faced. According to the Venice Commission this ‘supports the perception that the measures allowed by the Decree Law are *actually designed* and/or used to address (also) more general problems facing the Turkish authorities *as they see it*, not necessarily having a link to the management of the state of emergency [emphasis added].’ (Venice Commission 2017a, para 67). The Venice Commission also reiterated a substantive standard which applies despite the intentions of political branches, that structural (general) measures or individual measures with a permanent effect should be introduced in a normal manner and that there must always be a strict and genuine link between the reasons justifying the state of emergency and the measures taken through the emergency decree laws, which was absent in this case. Similarly, the Monitoring Committee felt the need to see through the government’s declared purposes: ‘If one combines all these events, facts and situations, one could easily conclude that the government might be pursuing an agenda of systematically acting against the HDP local elected representatives, using the mechanisms available in Turkish legislation, that is, by having recourse to abusive prosecutions’ (Monitoring Committee 2022, para 272). In this context, it deserves mentioning that a case is currently pending before the Constitutional Court concerning the complete ban of the HDP for its alleged connections with the Kurdistan Workers’ Party (PKK), further exemplifying the larger agenda of the central government to remove this political party from a political arena entirely (Monitoring Committee 2022, para 294).

Attacks on opposition-led local governments continued during COVID-19 as well both in Turkey and Hungary. Unlike Hungary (Kovács 2022), this time Turkey had not invoked the constitutional regime of emergency and the executive resorted to vast executive powers of the new presidential system (Çalı and Turkut 2022). The measures adopted in Hungary and Turkey during the pandemic as described below range from the ban of local initiatives, cutting of funds, and confiscation of profit-generating land. The practice of substituting elected local officials by unelected bureaucrats in Turkey as described above continued throughout the pandemic as well, however, that was not specifically justified by the new crisis.

Fundraising campaigns initiated by opposition mayors of Istanbul and Ankara for those harmed by the pandemic were swiftly blocked by the Minister of Interior circular stating that the municipalities were creating a state within the state. The central government froze the donations and never returned them.On the same day, Erdoğan government reintroduced the campaign on the national level (Savaşkan 2021, 219; San et al. 2021, Çalı and Turkut 2022, 256). In Hungary, with the justification of remedying consequences of the COVID-19 pandemic, certain taxes (e.g., vehicle tax, car parking fees, business tax) were suspended, while the opposition-led cities most affected with such cuts were also obliged to pay a higher solidarity tax. Despite being neutral, these measures disproportionately decreased revenues of certain local municipalities, especially the opposition-led capital. Messages that certain funds would decrease in case opposition won the capital had been voiced by Fidesz politicians already during the electoral campaign before the pandemic had created the new pretexts (Euronews 2021; Hajnal et al. 2021; Kovarek and Littvay 2022).

The most outstanding example of using COVID-related pretext comes from Hungary, and as in Turkey, targets a more vulnerable local actor—small city of Göd led by opposition. Thanks to the extraordinary powers under the Enabling Act, Government issued two emergency decrees on the same day on April 17, 2020, one allowing the forming of ‘special economic areas’ within the territories of municipalities with real estate transferred from the municipalities to the counties to ‘protect investments of national economic importance’, and the second creating such an economic zone in the city of Göd with a Samsung factory that is now paying the taxes to the Pest County (governed by Fidesz-KDNP). The decrees do not define criteria for changing status of territories to ‘special economic areas’ and the city had not been compensated for the loss of revenue (1/3 of the total). As Karsai observes, ‘[t]he decrees cannot be justified by any real economic steps, they simply change the ownership structure of valuable real estate and transfer the related taxation rights. [..] In the actual case the fake nature of the measures is crystal-clear, since Samsung does not even have to pay a forint less tax in the future; it just pays taxes to the County instead of the city of Göd.’ He finds further evidence for the ulterior motive of the central government when comparing Göd to other cities led by FIDESZ with similar factories and observed difficulties, that were not targeted. After testing this measure in an emergency context, the government submitted a general bill with a similar rule allowing the creation of free economic zones, now with the possibility of transferring anything above 5 billion forints, rather than 100 billion forints in the decree. This cements a broader version of the temporary emergency measure beyond the period of crisis (Karsai 2020).

This case is an illustrative example of using the COVID-19 pandemic as a pretext for undermining opposition-led subnational governments. However, the Constitutional Court when discussing this issue did not doubt the validity of the legislative aim presented in any way (Hungary Today, 2021). It stated that property meant social responsibilities and could be restricted, moreover, standards were even lower with municipality-held property, as it was meant for the fulfillment of public tasks, and not for free disposal. According to the Constitutional Court, free transfer of immovable properties belonging to non-tradable local government assets that are part of the national wealth to the county government was proportionate, given that the latter would fulfill public tasks in connection with that property (taxation, economic organization and administration). Thus, according to the decision, the constitutional guarantee on self-government autonomy was not violated. The Constitutional Court did set a constitutional requirement that such a transfer could not put the municipality in a situation in which it would not be able to perform assigned public tasks, however, there was no measure taken to ensure that this would be guaranteed. At no stage, did the Constitutional Court pay attention to institutional aspects of undermining local government specifically led by opposition due to abrupt deprivation of property besides the context of performing public tasks. Moreover, the Constitutional Court interpreted COVID-19 as a crisis with long-term effects beyond its immediate presence, which extended the pretextual potential of the health crisis even when the pandemic receded.^16^. The decision is among other politically sensitive cases, in which the deferential Court attempts to avoid embarrassing the government by merely declaring constitutional requirements of interpreting the law (Pozsár-Szentmiklósy 2020, Chronowvski 2021, Kazai 2022).

The above trend of undermining opposition-led local governments was facilitated through the help of crisis-related pretexts. While the pre-pandemic centralization measures already entailed risks of abuse, and centralization proceeded incrementally, the crisis related to the attempted coup (in Turkey) and COVID-19 significantly extended the possibilities. In these situations, crisis became a pretext that ‘legitimized’ more radical changes than previously tolerated, at the same time, the measures and its rationales diverged more. Declaration of an ‘economic zone’ leading to confiscation of profit-generating property only in the opposition-led city of Göd in no way affected the operation of a business or its employees despite the pretext that it was serving crisis-management. Similarly, adoption of the changes, that had been attempted and failed before, during post-coup emergency in Turkey to fight the crime of local government leaders also displayed publicly observable traits of pretext. In this case, pretext could also be evidenced through the inconsistency in the reasoning about the need for such a radical measure, as the previous rule already permitted substitution of elected officials, but by other elected, rather than government officers. Apart from that, the practical application of the rule, that mayors were removed exclusively from the opposition and that the measure enabled complete substitution of the pool of opposition mayors from one party, do add objective weight to the evidence of pretext. This highlights the relevance of assessing ex post selective/abusive application of a general rule for establishing ulterior motives. Traits of a pretext could also be observed when the mandate of opposition leaders were denied by SEC in Turkey, even though disqualification from elections was not considered by the same body before. Clear deviation from a norm established by the same body makes the stated goals suspect. The possibility of legally constructing pretext was also implied in the reports of such authoritative bodies as Venice Commission and Monitoring Committee in relation to Turkish bill on substitution of elected mayors with trustees noting that emergency was used as a pretext for adopting laws that could not have gathered enough support before. Thus, it is argued here that crisis-related radicalization of measures also meant more vivid divergence from stated aims (pretexts) to ulterior one of undermining opposition-led local governments.

The examples from both Turkey and Hungary also illustrated that subnational illiberalism is an incremental process in that measures get tested against the most vulnerable to be normalized for a more across-the-board application later. Indeed, the center in Turkey is using the measure almost exclusively against the region with highly polarized local politics ruled by the most marginalized political party HDP, which represents the Kurdish minority in Turkey. Following the same pattern, ineligibility to hold mandate has been claimed by SEC for opposition mayors after the 2019 elections, while just a year before that was not allowed in relation to opposition MPs in a national election, which would presumably cause more backlash in the center. Similarly, the most drastic measure in Hungary was tested against a small town, rather than the opposition-ruled capital. This observation that creeping subnational illiberalism starts with the weaker and more invisible to get normalized later is another insight into how subnational illiberal democracies work and should guide us in conceptualizing adequate responses to it.

### Untangling Playbook of Subnational Illiberalism

The cursory review of Latin American experience and in-depth analysis of the more recent European examples prepares the grounds for untangling the playbook of subnational illiberalism some shared across the two continents already, while diffusion of others could still be forthcoming. In the end, we are left with the following categories in the playbook summarized one by one below: abuse of (existing) supervisory and accountability mechanisms; generating of financial vulnerability; centralization (outright and indirect) and deconcentration. Each of these categories assemble various means evolving through application and reinterpretation of traditional rules pertaining to local government (e.g., supervisory and accountability measures, discretionary subsidies), as well as crisis-induced innovations (e.g., confiscation of profit-generating property, substitution of elected mayors by appointed trustees without court decisions).

#### Abuse of (Existing) Supervisory and Accountability Mechanisms

The abusive potential of supervisory mechanisms depends on the scope of review powers, namely whether sanctions can follow from such ambiguous grounds such as cost-effectiveness of local government decisions beyond concrete breaches of law under legality review (Turkey and failed attempt in Poland). Turkey is also a good example of vague supervision grounds, such as prohibition of ‘political decisions’ on the local level. In extreme situations, novel policies may get cancelled due to their contradiction to vague legal requirements not specifically foreseen in legislation, such as the impermissibility of creating state within a state in Turkey.

Whilst narrowly construed in Hungary and Poland, and in administrative tutelage framework in Turkey (requiring judicial confirmation), accountability mechanisms foresee a theoretical possibility of removing elected officials, which can be abused against political opponents in the local government. Apart from administrative tutelage framework in Turkey requiring a court approval for removing elected local officials, until 2016, the Turkish Constitution and law also allowed temporary removals of mayors due to ongoing criminal investigations before the court decision was made provided that the municipal council elected a new mayor. In the post-coup period in 2016, this was changed with regard to investigations on terrorism-related charges, and now at any stage of investigation, elected mayors can be substituted by centrally appointed trustees. Through this change, the traditional accountability mechanism as a means of attacking opposition-led local governments became superfluous. Without safeguards such as judicial supervision and substitution by other elected officials, such a possibility of removing elected mayors has now combined the weaknesses of prior rules directly contradicting basic tenets of democracy and in that way, representing a new phenomenon even for hybrid regimes. Awaiting the 2019 local election results the measure got even more radicalized by denying electoral mandate through Supreme Election Council without new investigations or prior disqualification from elections grounding the decisions in the decrees issued during the post-coup emergency that banned the elected leaders from public office. As the polish (failed) example also illustrates, abusive potential of supervisory and accountability mechanisms also depends on the composition and resulting independence of involved bodies.

Moreover, nation-wide network of supervisory bodies in Hungary among others can take steps with the assent of Treasury of State to suspend or withdraw a certain portion of support from the central budget. Apart from being an accountability measure, the same may affect the financial capacity of the local government, bridging to the next cluster of tactics.

#### Generating Financial Vulnerability of Local Government

Financial means can get controlled by the center through various measures such as changing calculation methodologies for transfers, non-automatic resource devolution, centrally controlled distribution of loans, off-budget complementary distributions etc. The measures listed above range from entailing the least discretion of the center, when the changed calculation methodology is universally applied for devolving resources, to the most discretionary schemes such as off-budget complementary distributions, which appeared as a direct means of favoritism and punishment. It must be noted from the outset that discretionary mechanisms of devolving resources become all the more problematic when own revenues constitute a small part of the total local budget.

Discretionary funding schemes such as conditional and task-based funding in Hungary and loan schemes from the center both in Hungary and Turkey are generally more readily available in unitary states. As best evidenced in Turkey, funds can be decreased indirectly and step-by-step by changing the composition of bodies that make decisions on loans subject to central control.

Somewhat emulating the tactic of removing certain revenues from a shareable pool in Latin America in new calculation methodologies, government in Hungary using the health crisis as a pretext removed certain geographic areas from the taxing mandate of the local authorities. This measure was easier to defend with the constitutional changes that had not differentiated local government property from other national property. Similar measure also exists within administrative tutelage system in Turkey, where properties of municipalities and of special provinces can be transferred to the Ministry of Interior, but in this case for the reasons of security. Claiming exceptional circumstances during crisis, the central government in Hungary also disproportionately reduced local revenue for opposition-led local governments in urban areas through combination of seemingly universal and neutral tax suspensions and contributions of a redistributive solidarity tax.

#### From Centralization (Outright and Indirect) to Deconcentration

Direct sectoral centralization has taken place in Hungary, Poland and Turkey and such examples are present in Latin American experience as well. Indirect ways such as changing composition of decision-making bodies (Hungary) or those supervising them (Poland) have also been used to turn locally controlled bodies intro centralized ones.

Prime example of parallel deconcentrated bodies in the form of a nationwide network come from Venezuela (even funded at the expense of subnational governments) to be emulated by Orbán in the new 2011 Constitution by introducing the network of deconcentrated county government offices. A similar administrative tutelage system is embedded in Turkey. Even without creating such horizontal networks, deconcentrated bodies in specific sectors may dilute and even override the overlapping local powers as shown by examples both from Turkey and Hungary.

## Conclusion

The analysis of European experience in light of Latin American examples produced general observations about the context and dynamics of applying the playbook discussed. Namely, the main tactics against opposition-led local governments are subtle, incremental and take a technical/legalist form, whilst they radicalize and become most vividly confrontational with crisis-related new pretexts. The subtle nature of regular recentralization is not surprising as current principles of local government in unitary states already contain elements that can be abused for illiberal purposes, for instance, the mechanisms of legal supervision and the possibility of sanctioning for the breach, even with dismissals and dissolutions. Despite such embedded weaknesses, popular support, culture and antecedent guarantees for local democracy matter in constraining the central ambitions, as confirmed by the Polish case. This culture was also visible in the Polish President’s veto that blocked retrogressive legislation even when the law was adopted by the Parliament dominated by his own party. As for more radical tactics originating during the crisis,  they were still incremental in that most vulnerable actors were targeted first. Then, as demonstrated, crisis-related measures got normalized for a potential across-the-board application characteristic to emergency and exceptional regimes in general (Dyzenhaus 2001, Agamben 2005; Sajó 2006, Grogan and Donald 2022, 478–479)*.*

Given the above points, the logic of subnational illiberalism is incremental, opting for subtle legalistic and technical means of weakening autonomy of local governments, whilst crisis may radicalize the measures and exemplify the ulterior purposes, as the law remains an instrument. The latter point was best illustrated by the Hungarian case of confiscating the profit-generating property only from an opposition-led city, and the Turkish cases of removing mayors and denial of democratic mandate through ex post disqualification. In these situations, both in Hungary and Turkey contradictions in justifications for the stated neutral goals were so serious that they amounted to publicly observable objective traits of impermissible ulterior motive, such as undermining of political opponents by generating financial vulnerability, depriving them of electoral office and of political victory. Such cases also raise a theoretical question whether phenomenon of pretext can be judicially constructed. That there is clear partisan distance between the rule-maker and the affected, often accompanied by selective application of the rule, makes such tactics of subnational illiberalism more appropriate to be seen as ‘suspect’. There are also normative reasons for doing so as preventing pursuance of ulterior motives against opposition-led local governments serves competitive political markets (Issacharoff and Pildes 1998).

There is a whole new conversation to be held considering the ample theoretical and practical problems of attributing bad faith to co-equal branches of government from the perspective of separation of powers. Despite being one of the firsts in constitutional law,^17^ the problem of imputing bad faith to co-equal branches, especially multi-member bodies such as Parliament, is still seen a marginal development. This is not surprising as judges are aware of retaliation risks likely to follow from such an exercise (Pozen 2016, Nelson 2008, Gardbaum 2016, Dworkin 1986; Farrell, 1992; Kent and Denning 2014, Sajó 2019, 372). As Uitz succinctly puts it ‘a strong presumption of constitutionality together with expectations of judicial deference’ requires particularly convincing evidence for finding of a pretext ‘on pain of a severe political backlash directed at the judiciary.’ (Uitz 2019, 474). Nevertheless, such evidence is sometimes still found (Helfer 2020, 227–229; Tsampi 2020, 135, Tan 2018, 113; Pech and Kochenov 2021, 30, 68–74; Venice Commission 2017b, para 47). The view that there can be reliable, judicially manageable standards on pretextual rulemaking is gaining more ground in the literature as well (Landau 2019, 2020; Fallon 2016). I argue here that this was the case with the discussed crisis-related measures in Hungary and Turkey.

Setting aside the implications of these observations for the judicial doctrine of pretext for now, the immediate goal of the article has been fulfilled by untangling subnational portion of illiberal playbook categorized as follows: abuse of (existing) supervisory and accountability mechanisms (e.g., legality review, court-approved dismissals of elected local officials); generating of financial vulnerability (e.g., changing composition of bodies giving out loans); outright or indirect centralization and deconcentration. Each of these categories assemble various means evolving through application and reinterpretation of traditional rules pertaining to local government, as well as crisis-induced innovations. One cannot but notice that the measures discussed above are more prone to abuse in autocratizing regimes such as Hungary, Poland and Turkey, where independent institutions such as courts, electoral authorities, national banks etc. have already been co-opted.

## Footnotes

1 European Charter of Local Self-Government 1985 https://rm.coe.int/168007a088

2 This is common in Hungary mainly through procedural tinkering with organizational rules governing city councils. For instance, such changes for Budapest city council increased the financial threshold for relevant committee review of financial transactions from 50 million to 5 billion forints.

3 For instance, in Paraguay, a mayor could be removed from office by the vote of an absolute majority of the National Congress, following a request by an absolute majority of councillors on ambiguous grounds of ‘poor performance of duties’.

4 During the presidency of Andres Pastrana, Colombia switched from percentage-based revenue sharing to inflation-adjusted fixed sum. New revenue classifications were introduced to exclude certain revenues from eligibility for transfers to subnational governments in Ecuador, Venezuela and Brazil, during the presidencies of Correa, Chávez and Cardoso, respectively.

5 By 2014, Poland was ranked amongst the ‘top ten’ countries within the EU Member States for its level of local autonomy.

6 In 2004–2005 urban planning issues became local responsibility in Turkey, however, soon this also fell under the authority of TOKI (the government-backed Housing Development Agency of Turkey), when large areas of irregularly settled land was nationalized for development projects according to changes in the law. TOKI also acquired the power to override local decisions. In 2011–2012, Ministry of Environment and Urbanisation was created and given the power to undertake the planning of urban transformation projects in places at risk of earthquakes or other disasters, which was broadly interpreted.

7 Slater and Simmons describe critical antecedents, as those which direct attention to ‘factors or conditions preceding a critical juncture [that] combine in a causal sequence with factors during a critical juncture to produce divergent long-term outcomes’.

8 Article 165.2 of the Constitution: ‘The self-governing nature of units of local government shall be protected by the courts’. Each local government unit that is allegedly affected by a piece of legislation is allowed to file a complaint with the Constitutional Court.

9 Turkish constitutional court has had scarce case law on the principles of local self-government. In addition, local authorities do not have standing to sue in the Constitutional Court to claim unconstitutionality of laws. Local authorities can go to ordinary court system, which can refer the case to the Constitutional Court. However, this has never happened.

10 While the government itself upon the motion of County Government Offices may address the constitutional court for review of municipality decrees, judicial remedies for the municipalities themselves are very limited. As a rule, local authorities may not apply directly to the Constitutional Court except in case of conflict with another authority concerning their respective responsibilities, or if they act as private entities, or indirectly through the mechanism of preliminary ruling.

11 According to the Municipality Law (Act No. 5393), the purpose of the inspection in the municipalities is to perform an impartial analysis and comparison of the services delivered by the local authority, taking certain performance standards as a reference point. As a result of the inspections, a report is prepared containing findings to be submitted to the relevant authorities, with a view to avoiding the 'failures' detected in the activities of the municipality and improving the quality and efficiency of the local services,

12 For example, the Municipal Council of Sur, adopted the use of languages in addition to Turkish in its provision of services in 2007, which the Ministry of Interior regarded as a political decision and addressed the Council of State to terminate the activities of the municipal council.

13 In February 2020, representatives of three ministries (one from each) were added as members in the the steering council of Transport Coordination Centres (UKOME)—authorities which take decisions on urban transportation systems, parking spots, city traffic plans etc. Although the head of that body is the mayor, with the new composition the metropolitan municipalities such as Ankara and Istanbul will no longer have the initiative in decision-making.

14 In 2018, a new institution—Metropolitan Council for Public Investments was created based on a government decree specifically affecting the capital and all development decisions in Budapest. The Council consists of government and city representatives with the Prime-Minister as a chair, however, the decision is rendered unanimously, which in practice grants veto power to the center.

15 The state referred to a general competence under Article 79, paragraph 1 of the Constitution to ‘execute all the functions to ensure the fair and orderly conduct of the elections from the beginning to the end of polling, carry out investigations and take final decisions on all irregularities, complaints, and objections concerning the elections during and after the polling, and verify the election returns of the members of the Turkish Grand National Assembly.’.

16 Constitutional Court Decision 8/2021. (III. 2.) AB http://public.mkab.hu/dev/dontesek.nsf/0/EE964910BAF66F71C125856E005C1D98?OpenDocument&fbclid=IwAR0mmsKAqbDcjMO7HSmpCFlZ_Pne3Jj9Q33LZDBzMjyQksivoTV3Q-zjehY

17 As Chief Justice Marshall stated already in 1819 'Should Congress, in the execution of its powers, adopt measures which are prohibited by the constitution; or should Congress, under the pretext of executing its powers, pass laws for the accomplishment of objects not entrusted to the government; it would become the painful duty of this tribunal, should a case requiring such a decision come before it, to say that such an act was not the law of the land.' McCulloch v. Maryland, 17 US 316 (1819).
